# Comparison of mini percutaneous nephrolithotomy and standard percutaneous nephrolithotomy for renal stones >2cm: a systematic review and meta-analysis

**DOI:** 10.1590/S1677-5538.IBJU.2021.0347

**Published:** 2021-10-08

**Authors:** Pengfei Qin, Dong Zhang, Ting Huang, Li Fang, Yue Cheng

**Affiliations:** 1 School of Medicine Ningbo University Ningbo Zhejiang China School of Medicine, Ningbo University, Ningbo, Zhejiang, China;; 2 Department of Urology The Center for Uro-nephrological Diseases Ningbo First Hospital Ningbo Zhejiang China Department of Urology, The Center for Uro-nephrological Diseases, Ningbo First Hospital, Ningbo, Zhejiang, China;

**Keywords:** Nephrolithotomy, Percutaneous, Kidney Calculi, Meta-Analysis as Topic

## Abstract

**Background:**

The purpose is to compare the efficacy and safety of mini percutaneous nephrolithotomy (mini-PCNL) versus standard percutaneous nephrolithotomy (standard-PCNL) in patients with renal stones >2cm.

**Materials and Methods:**

A systematic literature search was conducted in PubMed, Web of Science, Scopus, and the Cochrane Library databases to identify relevant studies before March 8, 2021. Stone-free rate (SFR), operation time, fever rate, hemoglobin drop, blood transfusion rate, and hospitalization time were used as outcomes to compare mini-PCNL and standard-PCNL. The meta-analysis was performed using the Review Manager version 5.4.

**Results:**

Seven randomized controlled trials were included in our meta-analysis, involving 1407 mini-PCNL cases and 1436 standard-PCNL cases. Our results reveal that, for renal stones >2cm, mini-PCNL has a similar SFR (risk ratio (RR)=1.01, 95% confidence interval (CI): 0.98 to 1.04, p=0.57) and fever rate (RR=1.22, 95% CI: 0.97-1.51, p=0.08). Standard-PCNL was associated with a significantly shorter operating time (weighted mean difference (WMD)=8.23, 95% CI: 3.44 to 13.01, p <0.01) and a longer hospitalization time (WMD=-20.05, 95% CI: -29.28 to -10.81, p <0.01) than mini-PCNL. Subgroup analysis showed hemoglobin drop and blood transfusion for 30F standard-PCNL were more common than mini-PCNL (WMD=-0.95, 95% CI: -1.40 to -0.50, p <0.01; RR=0.20, 95% CI: 0.07 to 0.58, p <0.01).

**Conclusion:**

In the treatment of >2cm renal stones, mini-PCNL should be considered an effective and reliable alternative to standard-PCNL (30F). It achieves a comparable SFR to standard-PCNL, but with less blood loss, lower transfusion rate, and shorter hospitalization. However, the mini-PCNL does not show a significant advantage over the 24F standard-PCNL. On the contrary, this procedure takes a longer operation time.

**Trial registration:**

This meta-analysis was reported consistent with the PRISMA statement and was registered on PROSPERO, with registration number 2021CRD42021234893.

## INTRODUCTION

Percutaneous nephrolithotripsy (24-30F) remains the standard procedure for treating large renal calculi ( 1 ). While achieving high SFR, it also has many drawbacks such as bleeding, postoperative pain, and a long recovery period due to its large access tract ( 2 ), so the mini percutaneous nephrolithotripsy (14-22F) with a smaller tract size came into being. It has been more than 20 years since Jackman et al. ( 3 ) and Helal et al. ( 4 ) first reported the application of mini-PCNL in pediatric surgery. Although numerous studies have been conducted on comparing the two types of percutaneous nephrolithotripsy, the debate on which one is better continues, and the main point of conflict is the difference in SFR and incidence of postoperative complications ( 5 - 8 ). In the treatment of renal stones >2cm, retrograde intrarenal surgery (RIRS) and extracorporeal shock wave lithotripsy (ESWL) seem not to be competitive enough compared with PCNL, so can mini-PCNL, which is more minimally invasive, be used as a substitute to standard-PCNL in such cases? Scholars ( 9 - 11 ) have systematically reviewed the comparison of percutaneous nephrolithotripsy with different tract sizes. However, the quality of the included evidence was poor, and more reliable data from randomized controlled trial (RCT) studies are needed. Furthermore, there was no meta-analysis comparing standard-PCNL and mini-PCNL in patients with large kidney stone burdens. Therefore, our focus is on comparing surgical procedures for renal stones >2cm, and updated RCT studies in recent years were added, including some high-quality large multicenter RCT studies such as Zeng et al. ( 12 ). Efficacy and safety of the two surgical procedures in renal stones >2cm were compared, and subgroup analyses were performed to derive a more optimal recommendation for clinical practice.

## MATERIALS AND METHODS

### Search strategy

Registration for this study was conducted on PROSPERO, with registration number 2021CRD42021234893. Two independent authors conducted separate searches in PubMed, Web of Science, Scopus, and the Cochrane Library databases to identify relevant studies before March 8, 2021. Only articles published in English were selected. The key words we used in the search were “mini percutaneous nephrolithotomy” OR “mini-PCNL” OR “miniperc” OR “MPCNL” OR “minimally invasive percutaneous nephrolithotomy” AND “standard percutaneous nephrolithotomy” OR “standard PCNL”. We also searched for relevant systematic reviews and references to identify any omitted studies ( 13 ). The articles which meet our inclusion criteria were selected based on their titles and abstracts.

### Selection of studies

The literature selection was performed independently by two authors according to Preferred Reporting Items for Systematic Reviews and Meta-Analyses (PRISMA) guidelines ( 14 ). Disagreement was resolved by consensus or arbitrated by a senior author. Inclusion and exclusion criteria were specified before our search. The inclusion criteria were as follows: ( 1 ) available RCT studies; ( 2 ) patients with renal stones >2cm; ( 3 ) studies that compared mini-PCNL with standard PCNL; ( 4 ) reporting at least one of the following outcomes: SFR, operation time, hospitalization time, hemoglobin drop, blood transfusion, fever. Exclusion criteria were composed of: ( 1 ) pediatric patients (<18 years old); ( 2 ) super-mini/ultra-mini/micro percutaneous nephrolithotomy (<14F); ( 3 ) case reports, conference abstracts, editorials, reviews, animal experiments, and letters.

### Data extraction

All the articles included were read, and the relevant data from the articles were also extracted on a standard form by two reviewers. The primary analyzed outcome was SFR. The secondary outcomes were operation time, hospitalization time, hemoglobin drop, blood transfusion, and fever. For some continuous variable data reported using the median and the first and third quartiles, we converted them into sample mean and standard deviation according to the method improved by Luo et al. ( 15 ) and Wan et al. ( 16 ) to pool results in a consistent format.

### Quality assessment

The level of evidence (LE) for included studies was assigned according to the criteria provided by Oxford Centre for Evidence-Based Medicine ( 17 ). The risk of bias assessment for these RCT studies was based on the Cochrane Systematic Reviews Manual, in which studies were evaluated in seven aspects (allocation concealment, random sequence generation, blinding of participants and personnel, selective reporting, incomplete outcome data, blinding of outcome assessment and other bias). Any discrepancy was resolved by consensus.

### Statistical analysis

The related data analysis was performed using the Review Manager version 5.4 (Cochrane Collaboration, UK). Risk ratio (RR) and weighted mean difference (WMD) were used to evaluate the dichotomous variables and continuous parameters, respectively. Both types of data were reported with 95% confidence intervals (CI). The Chi-square test and I^2^ statistic were used to calculate statistical heterogeneity among included studies. When I^2^ <50%, fixed-effect models were used, and random-effect models were applied for the meta-analysis when I^2^>50%. In addition, the pooled effects were assessed by the Z test. For the result of data analysis, a P <0.05 can be considered statistically significant. Subgroup analysis was performed on all outcomes by dividing the standard-PCNL group into 30F and 24F groups. Forest plots were drawn to present the results of the meta-analysis. In order to evaluate the stability of the meta-analysis results, a sensitivity analysis was performed by leave-one-out cross validation.

## RESULTS

### Study selection

**Figure 1:**
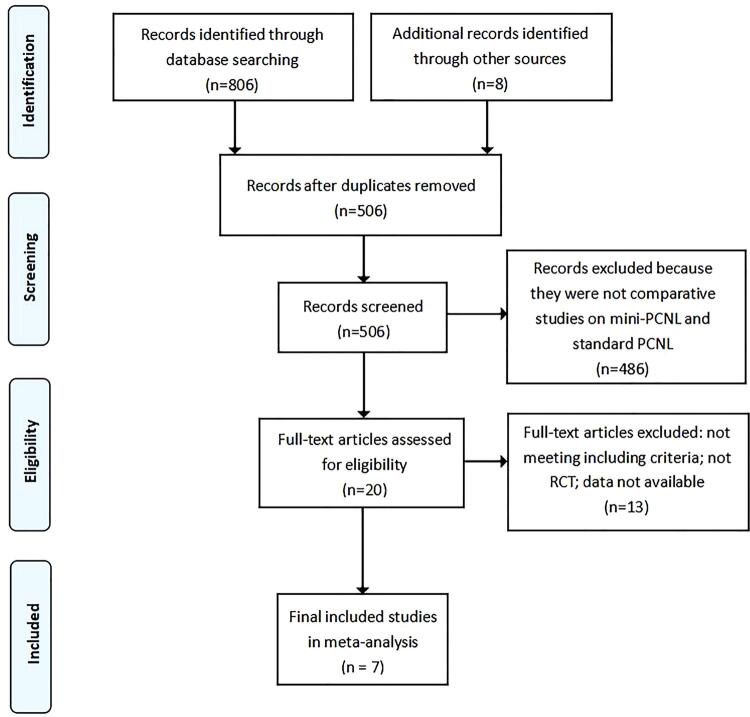
Flow diagram of studies selection process.

The study search process and results are shown in Figure-1 . A total of 814 studies were collected using the search strategy mentioned above, and 7 studies were finally considered eligible after the exclusion ( Table- 1). All 7 studies are randomized controlled trials, including 1407 mini-PCNL cases and 1436 standard-PCNL cases ( 5 , 6 , 13 , 18 - 21 ). All of the included studies compared mini-PCNL with standard-PCNL for patients with kidney stones larger than 2cm.

**Table 1:** Summary of comparative studies.

Study	Country	Study period	Study design	LE	Cases, n		Definition of SFR
					Mini	Standard	
Güler et al. ( 18 )	Turkey	2016.01-2017.04	RCT	2b	51	46	complete clearance of stones
Kandemir et al. ( 5 )	Turkey	2016.11-2018.09	RCT	2b	76	72	complete clearance of stones or with residual fragments <4 mm
Wu et al. ( 19 )	China	2014.03-2015.07	RCT	2b	114	114	complete clearance of stones or with residual fragments <4 mm
Zeng et al. ( 12 )	China	2016.01-2019.08	RCT	2b	978	966	complete clearance of stones or <4mm asymptomatic, noninfectious, and non-obstructive residual stones at 1 month after the removal of the J-J stent
Sakr et al. ( 6 )	Egypt	2010.09-2013.12	RCT	2b	75	75	complete clearance of stones or with residual fragments <4 mm
Cheng et al. ( 20 )	China	2004.05-2007.12	RCT	2b	69	111	complete clearance of stones or with residual fragments <4 mm
Song et al. ( 21 )	China	2008.08-2009.08	RCT	2b	30	30	complete clearance of stones or with residual fragments <4 mm

### Characteristics and quality of the included studies

**Figure 2:**
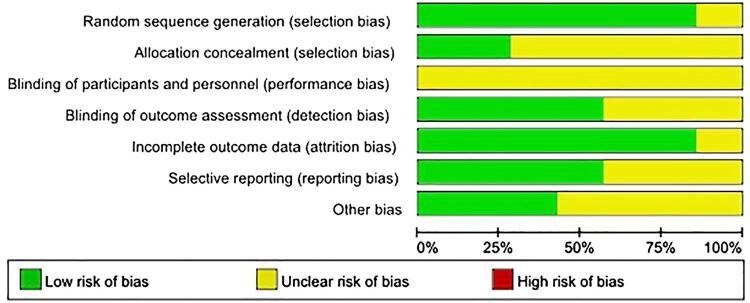
Overall quality assessment for the included articles.

The baseline characteristics of the 7 studies such as age, stone burden and tract size are shown in Table-2 . Actual surgical procedures varied in terms of access sheath size, dilator, nephroscope size and type of lithotriptor. In all the studies included in this meta-analysis, the access sheath size of standard-PCNL was 30F or 24F. The level of evidence of the included literature is described in Table-1 , and the quality of the studies was assessed by Cochrane’s risk of bias tool in Figure-2 . There is some “unclear risk of bias” in the assessment results because some literature is inadequate in some trial details.

**Table 2:** Baseline characteristics of included studies.

Study	Mean age		Mean stone burden		Access sheath size		Dilator		Nephroscope size		Lithotripsy	
	Mini	Standard	Mini	Standard	Mini	Standard	Mini	Standard	Mini	Standard	Mini	Standard
Güler et al. ( 18 )	46.9 ± 13.7	47.4 ± 13.9	38.7 ± 13.1mm	42.8 ± 22.5mm	16.5/20F	30F	AD	BD/ AD	12F	26F	Laser	Pneumatic and ultrasonic
Kandemir et al. ( 5 )	47.0 ± 13.9	46.7 ± 14.2	32.6 ± 8.1mm	33.1 ± 10.9mm	16.5/20F	30F	AD	BD/ AD	12/14F	26F	Laser	Pneumatic, ultrasonic, laser
Wu et al. ( 19 )	47.6 ± 8.2	48.1 ± 7.9	3.4 ± 1.0cm	3.3 ± 1.1cm	16F	24F	FD	AD	8/9.8F	20.8F	Ultrasonic	Ultrasonic
Zeng et al. ( 12 )	51.0 (43.0, 59.0)^a^	51.0 (44.0, 60.0)^a^	29.0 (23.0, 35.0)mm^a^	29.0 (25.0, 35.0)mm^a^	18F	24F	FD	FD	12F	20.8F	Pneumatic, ultrasonic, laser	Pneumatic, ultrasonic, laser
Sakr et al. ( 6 )	43.8 ± 9.5	40.2 ± 8.3	2.7 ± 0.2cm	2.6 ± 0.6cm	16.5F	30F	TMD	TMD	12F	26F	Pneumatic	Pneumatic
Cheng et al. ( 20 )	37.2	39.6	9.54cm^2^	9.62cm^2^	16F	24F	TMD	TMD	8/9.8F	20.8F	Pneumatic	Pneumatic and ultrasonic
Song et al. ( 21 )	NA	NA	8.57 ± 2.2cm^2^	8.65 ± 2.0cm^2^	16F	24F	FD	FD + TMD	NA	24F	Laser	Pneumatic and ultrasonic

**FD** = fascial dilators; **TMD** = telescoping metal dilators; **AD** = Amplatz dilators; **BD** = balloon dilators; **NA** = not available

^a^ Data are presented as median (first quartile, third quartile)

### Meta-analysis outcomes

#### SFR

**Figure 3:**
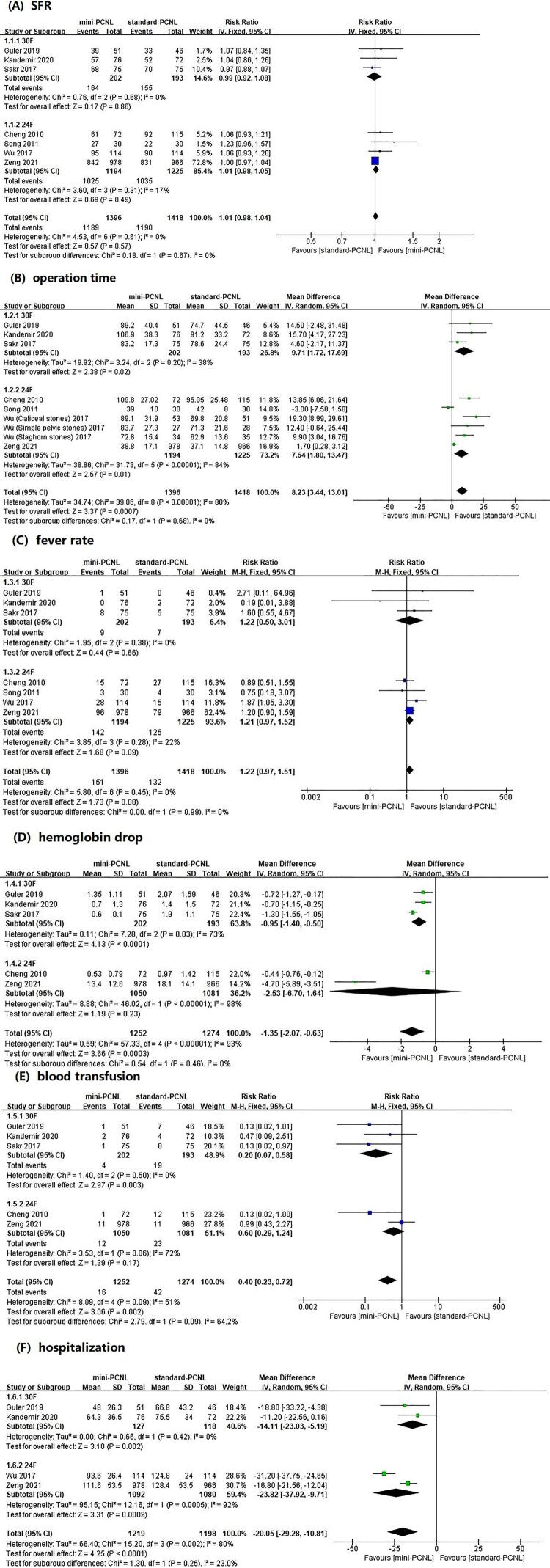
Forest plots and meta-analysis. (a) SFR, (b) operation time, (c) fever rate, (d) hemoglobin drop, (e) blood transfusion, (f) hospitalization.

Data on SFR were available in all seven studies, and the pooled results showed no significant difference in SFR between mini-PCNL and standard-PCNL (RR=1.01, 95% CI: 0.98 to 1.04, p=0.57, Figure-3 A ). The result of subgroup analysis also showed no difference between the 30F subgroup and the 24F subgroup and the mini-PCNL group (RR=0.99, 95% CI: 0.92 to 1.08, p=0.86; RR=1.01, 95% CI: 0.98 to 1.05, p=0.49). Mild heterogeneity was detected in the 24F subgroup (I^2^ =17%), while there was no heterogeneity in comparison of mini-PCNL and standard-PCNL (I^2^ =0%).

#### Operation time

The pooled results showed that the standard-PCNL group was associated with a significantly shorter operating time than the mini-PCNL group (WMD=8.23, 95% CI: 3.44 to 13.01, p <0.01, Figure-3 B ) while high heterogeneity (I^2^ =80%) was observed. The results of subgroup analysis showed that both the 30F subgroup and the 24F subgroup were superior to the mini-PCNL group in terms of operation time (WMD=9.71, 95% CI: 1.72 to 17.69, p=0.02; WMD=7.64, 95% CI: 1.80 to 13.47, p=0.01). The high heterogeneity was mainly detected in the 24F subgroup (I^2^ =84%).

## Fever

With data extracted from all seven studies, fever rate was higher with mini-PCNL. However, no statistical difference was found in fever rate between the mini-PCNL group and the standard-PCNL group according to this meta-analysis (RR=1.22, 95% CI: 0.97-1.51, p=0.08, Figure-3 C ). The result of subgroup analysis showed no statistical difference between the 30F subgroup and the 24F subgroup versus the mini-PCNL group (RR=1.22, 95% CI: 0.50 to 3.01, p=0.66; RR=1.21, 95% CI: 0.97 to 1.52, p=0.09), with mild heterogeneity in the 24F subgroup (I^2^ =22%).

## Hemoglobin drop and blood transfusion

Overall, the comparison of hemoglobin drop and blood transfusion rate between the mini-PCNL group and the standard-PCNL group showed significant differences (WMD=-1.35, 95% CI: -2.07 to -0.63, p<0.01, Figure-3 D ; RR=0.40, 95% CI: 0.23 to 0.72, p <0.01, Figure-3E ). Compared with the mini-PCNL group, the 30F subgroup was associated with a greater hemoglobin drop and higher blood transfusion rate (WMD=-0.95, 95% CI: -1.40 to -0.50, p <0.01; RR=0.20, 95% CI: 0.07 to 0.58, p <0.01). However, the hemoglobin drop and blood transfusion rate in the 24F subgroup was not significantly different from that in the mini-PCNL group (WMD=-2.53, 95% CI: -6.70 to 1.64, p=0.23; RR=0.60, 95% CI: 0.29 to 1.24, p=0.17). In the subgroup analysis of hemoglobin drop, heterogeneity was high in both 30F and 24F subgroups (I^2^ =73%; I^2^ =98%) while in blood transfusion rate, only the 24F subgroup showed high heterogeneity (I^2^ =72%).

### HOSPITALIZATION

Data on hospitalization were available in four studies. The pooled results and subgroup analysis results indicated that the mini-PCNL group was associated with a significantly shorter hospitalization (WMD=-20.05, 95% CI: -29.28 to -10.81, p <0.01, Figure-3 F ; 30F: WMD=-14.11, 95% CI: -23.03 to -5.19, p <0.01; 24F: WMD=-23.82, 95% CI: -37.92 to -9.71, p <0.01). High heterogeneity was detected in the 24F subgroup (I^2^ =92%).

### SENSITIVITY ANALYSIS

A sensitivity analysis was performed by leave-one-out cross validation for some outcome indicators with high statistical heterogeneity. The analysis results showed no significant decrease in heterogeneity in operation time or hemoglobin drop after articles were sequentially removed. Study reported by Wu et al. ( 19 ) were considered the main sources of heterogeneity in hospitalization, and the result remained unchanged after its removal. Of note, in the analysis of fever rate, the results showed a significantly higher fever rate with mini-PCNL when a study reported by Cheng et al. ( 20 ) was removed.

## DISCUSSION

Mini-PCNL appears to be an increasingly popular procedure for the treatment of renal stones. However, whether it can be superior to standard-PCNL regarding efficacy and safety is still under debate worldwide ( 5 - 8 ). A study by Deng et al. ( 11 ) revealed a significantly higher SFR in standard-PCNL than the mini-PCNL in adult patients with <2cm renal stones, while no statistical difference was found between the two procedures in patients with >2cm renal stones. In their study, no other outcome was analyzed according to the stone size, nor have they been reported in the published literature ( 10 , 11 ). Therefore, it is necessary to compare the safety and efficacy of these two procedures in these specific cases with renal stones >2cm. Only RCTs were included to ensure the reliability of the conclusions, especially the study by Zeng et al., which is of great significance ( 12 ). It is generally believed that the tract size of standard-PCNL is 24F-30F, and that of mini-PCNL is 14F-22F ( 22 ). Ultramini-PCNL and micro-PCNL should not be comparable with standard-PCNL in terms of operation indication (especially >2cm stone), and they are not intended to replace standard-PCNL but to compete with ESWL and RIRS. Therefore, the studies about ultramini-PCNL (11-13F) and micro-PCNL (4.8-10F) were not included in this meta-analysis.

In the present study, the SFR achieved by mini-PCNL was similar to that by standard-PCNL, although the definition of the SFR in these studies was slightly different. This result was in accordance with that of Zhu et al. ( 10 ). Notably, no significant differences in SFR between the 30F subgroup and the mini-PCNL group were found, unlike in the reviews published by Deng et al. ( 11 ), where the former has a higher SFR. This proves that mini-PCNL has been non-inferior to standard-PCNL in one-session SFR. Moreover, a study by Cheng et al. revealed that mini-PCNL even achieved a better SFR than standard-PCNL in cases with multiple calyceal stones. This can be caused by using a narrower ureteroscope which can help us reach different calyces more easily ( 20 ).

Standard-PCNL shows significant advantages regard to operation time. However, there was a high degree of heterogeneity among the included studies, which could be attributed to the differences between surgical protocols and differences in the definition of operative time. Different types of lithotripsy modalities also differ in stone fragmentation efficiency. Laser lithotripsy has become the current mainstream modality and is favored by surgeons, which is largely due to its high efficiency. Compared with pneumatic lithotripsy and ultrasonic lithotripsy, it may shorten the operation time for patients with a large stone burden. In the included studies, we found that several types of lithotripsy were often used together in standard-PCNL, whereas a single lithotripsy modality was used in mini-PCNL, which could lead to bias. Two main factors make mini-PCNL takes longer. On the one hand, the vision of mini-nephroscope surgery is worse, which makes the operation more complicated; on the other hand, in order for the stone to pass through a mini tract, surgeons have to break the stones into smaller pieces, which also significantly prolongs the operation time. Moreover, recent studies have shown that supine position was associated with lower operative time in standard-PCNL and mini-PCNL than other positions ( 23 , 24 ). However, there is still no consensus on its efficacy and the incidence of complications. Xu et al. found that the trend towards metabolic acidosis was more evident as the irrigation time went by during mini-PCNL compared with standard-PCNL ( 25 ). Surgeons should keep in mind that the longer a patient spends under general anesthesia, the more postoperative complications and the slower recovery ( 26 ).

Mini-PCNL resulted in a higher rate of postoperative fever, though not statistically significant. Still, the potential fever risk is worth noting. The interspace between scope and access sheath is very important. As the diameter of the access sheath is decreased, the absolute space for irrigation outflow will also be reduced, which may lead to a higher renal pelvic pressure (RPP) and absorption of irrigation fluid ( 27 ). Infection can also result from broken stones containing endotoxins and bacteria, and thus even if the urine culture is negative before surgery, patients may still get a fever after surgery. In addition, Wu et al. found that cumulative time >60s with RPP >30mmHg will significantly increase the incidence of fever. Therefore, prevention of sepsis may be achieved by ensuring RRP remains <30mmHg during operation and indwelling the drainage tube after the operation ( 19 ).

The establishment of the access tract is considered the leading cause of PCNL blood loss, in which the size of the tract is a crucial factor ( 10 ). Both hemoglobin drop and transfusion rate were found lower in mini-PCNL compared to the 30F PCNL. Unlike the previous study ( 11 ), mini-PCNL showed a similar blood transfusion rate as standard-PCNL ( 24 F) in this meta-analysis, which is an impressive result. This may be attributed to the large kidney stone burden, and the bleeding was more severe when dealing with large stones, even if using a mini tract. The nephroscope and access sheath are often prized by the surgeon to reach different calyces, resulting in severe renal damage and bleeding. Despite using a smaller tract, PCNL is still a procedure performed through a non-natural orifice, meaning that the risk of bleeding can only be minimized but not eliminated.

Only four studies evaluated hospitalization time, which can increase the risk of bias. Published studies seem to have concluded that patients undergoing mini-PCNL have significantly shorter hospitalization ( 5 , 13 , 18 , 19 ). This may be explained by the higher tubeless rate, more minimal renal trauma and less postoperative pain in mini-PCNL.

According to the results of this meta-analysis, 24F standard-PCNL has the same SFR as mini-PCNL, with similar blood loss, but with a shorter operation time than mini-PCNL. It seems that 24F standard-PCNL is a better choice for the treatment of >2cm kidney stones, which appears to improve safety without compromising efficacy. In fact, the 24F PCNL is being favored by more and more urologists around the World, because it can greatly reduce the complications caused by the large tract. Encouragingly, mini-PCNL (<24F) is evolving rapidly to achieve improved efficacy while retaining the safety benefits of mini-PCNL ( 28 , 29 ). Smaller tract sizes, better efficacy, and lower complication rates will surely be achieved over time.

There were some limitations in this meta-analysis. First, the tract sizes of standard-PCNL used in included studies were only 30F and 24F, lacking data of 26F and 28F, which may increase the risk of selective bias. Second, high heterogeneity was detected among some studies, which can partly influence the accuracy of our study. Although a sensitivity analysis was performed, some of the heterogeneity was difficult to explain. Third, some other complications, such as postoperative pain, were not evaluated in this meta-analysis due to the lack of reports in the included studies. Fourth, relatively few studies were included in our meta-analysis because retrospective studies and case-control studies were excluded.

## CONCLUSIONS

For the treatment of >2cm renal stones, mini-PCNL should be considered an effective and reliable alternative to 30F standard-PCNL. It achieves a comparable SFR to the latter, but with less blood loss, lower transfusion rate and shorter hospitalization. However, the mini-PCNL does not show a significant advantage over the 24F standard-PCNL. On the contrary, this procedure takes a longer operation time. Of note, the relatively long operation time and potential risk of fever associated with mini-PCNL should be taken seriously. Further research involving more high-quality evidence is necessary to support and supplement this conclusion.
